# ZSCAN4 facilitates chromatin remodeling and promotes the cancer stem cell phenotype

**DOI:** 10.1038/s41388-020-1333-1

**Published:** 2020-06-07

**Authors:** Benjamin A. Portney, Michal Arad, Aditi Gupta, Robert A. Brown, Raju Khatri, Phyo Nay Lin, Andrea M. Hebert, Kristen H. Angster, Lorna E. Silipino, W. Alex Meltzer, Rodney J. Taylor, Michal Zalzman

**Affiliations:** 1 Department of Biochemistry and Molecular Biology, University of Maryland School of Medicine, Baltimore, MD 21201 USA; 2 Department of Otorhinolaryngology—Head and Neck Surgery, University of Maryland School of Medicine, Baltimore, MD 21201 USA; 3 Marlene and Stewart Greenbaum Cancer Center, University of Maryland School of Medicine, Baltimore, MD 21201 USA; 4 The Center for Stem Cell Biology and Regenerative Medicine, University of Maryland School of Medicine, Baltimore, MD 21201 USA

**Keywords:** Target identification, Chromatin remodelling, Cancer stem cells

## Abstract

Cancer stem cells (CSCs) are cells within tumors that maintain the ability to self-renew, drive tumor growth, and contribute to therapeutic resistance and cancer recurrence. In this study, we investigate the role of Zinc finger and SCAN domain containing 4 (ZSCAN4) in human head and neck squamous cell carcinoma (HNSCC). The murine *Zscan4* is involved in telomere maintenance and genomic stability of mouse embryonic stem cells. Our data indicate that the human *ZSCAN4* is enriched for, marks and is co-expressed with CSC markers in HNSCC. We show that transient ZSCAN4 induction for just 2 days increases CSC frequency both in vitro and in vivo and leads to upregulation of pluripotency and CSC factors. Importantly, we define for the first time the role of ZSCAN4 in altering the epigenetic profile and regulating the chromatin state. Our data show that ZSCAN4 leads to a functional histone 3 hyperacetylation at the promoters of OCT3/4 and NANOG, leading to an upregulation of CSC factors. Consistently, ZSCAN4 depletion leads to downregulation of CSC markers, decreased ability to form tumorspheres and severely affects tumor growth. Our study suggests that ZSCAN4 plays an important role in the maintenance of the CSC phenotype, indicating it is a potential therapeutic target in HNSCC.

## Introduction

Embryonic stem (ES) cells and cancer cells have unlimited capacity for self-renewal and share many properties, including gene expression networks. The naïve “stemness” state in ES cells is maintained by the core pluripotency master regulators OCT3/4, NANOG, and SOX2 [1, 2]. Previous studies have demonstrated that, similar to ES cells, cancer cells can harness these pluripotency factors for survival, inhibition of differentiation [3, 4], and self-renewal [5–7]. Indeed, combinations of the core pluripotency factors have been shown to reprogram cells back to an “ES cell like state”, known as induced pluripotent stem cells (iPSC) [8].

Many embryonic factors have emerged as key regulators of cancer stem cells (CSCs) and thus, therapeutic targets. CSCs, or tumor-initiating cells, have unlimited capacity to self-renew, and recapitulate all cell types within the tumor from a single cell [5, 7, 9–12]. CSCs are the suggested driving force of tumorigenicity, contributing to an aggressive phenotype and tumor recurrence [12, 13]. Much like other embryonic factors, the human *ZSCAN4* has been proposed to have significance in cancer [14, 15]. However, to date, the function of human ZSCAN4 or how it exerts its effects remains unknown.

The murine m*Zscan4* gene cluster is transiently expressed in mouse embryonic stem (mES) cells [16] and 2-cell stage embryos [17, 18]. In mES cells, m*Zscan4* regulates telomere maintenance and genomic stability [16]. It was further shown to restore mES cell developmental potency [19], replace c-Myc, and to facilitate the reactivation of early embryonic genes during generation of iPSC [20]. In combination with the core pluripotency factors, m*Zscan4* promotes the generation of iPSC [21]. Additional reports suggest that ZSCAN4 expression positively correlates with chromatin de-repression [22].

ES cells and cancer cells are characterized by open and permissive chromatin signatures, enriched in active histone marks [23–27]. In this research, we studied the role of human ZSCAN4 in cancer. Our data suggest a novel and unexpected role for ZSCAN4 in marking and facilitating the CSC phenotype. We show that ZSCAN4 is transiently expressed in head and neck squamous cell carcinoma (HNSCC) cell lines and is enriched in and marks CSCs. We show that ZSCAN4 induction leads to a significant increase in CSC frequency both in vitro and in vivo. Our data further reveal that ZSCAN4 interacts with the core pluripotency gene promoters and facilitates a functional histone hyperacetylation of histone H3, which in turn results in an upregulation of CSC markers. Conversely, ZSCAN4 depletion leads to downregulation of CSC markers, a reduction in open chromatin marks, a reduced ability to form tumorspheres in vitro, and severely affects the ability of HNSCCs cells to form tumors in vivo. Overall, our studies suggest ZSCAN4 plays a critical role in the maintenance of HNSCC cancer stem cells.

## Results

### ZSCAN4 is enriched in tumorspheres

**Fig. 1:**
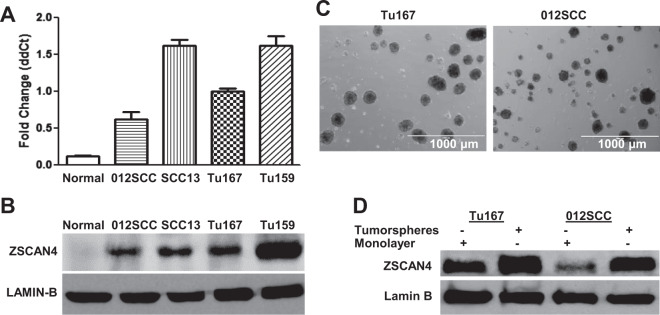
ZSCAN4 is expressed in HNSCC and is upregulated in tumorspheres. **a** ZSCAN4 is expressed in HNSCC cell lines, as shown by qPCR and by **b** immunoblot analyses, whereas normal human tonsil primary control cells from four different donors are negative. Error bars indicate S.E.M. **c** Representative phase contrast images of tumorspheres in WT HNSCC cell lines Tu167 and 012SCC. Scale bar indicates 1000 µm **d** immunoblot assays indicate that ZSCAN4 expression is enriched for in tumorspheres compared with attached cells in complete medium (monolayer).

To study the human *ZSCAN4* gene, we first sought to assess the expression of *ZSCAN4* by screening a panel of HNSCC cell lines (012SCC, SCC13, Tu167, Tu159) using quantitative reverse transcription PCR (qRT-PCR; Fig. 1a) and immunoblot analysis (Fig. 1b). Our data indicate ZSCAN4 is expressed in HNSCC cells, while the control human primary tonsillar cells are negative.

CSCs have been identified in HNSCC [9, 12, 13], contributing to cancer aggressiveness and cancer recurrence. Many stem cell factors are enriched for in cancer and are highly expressed in CSCs, highlighting their importance for prognostic prediction [28]. CSCs can be enriched for by their ability to form spheroids (tumorspheres) in non-adherent culture conditions in defined medium [10, 29]. Therefore, we utilized the tumorsphere assay in Tu167 and 012SCC cells and assessed the effect on ZSCAN4. Following 8 days in culture, tumorspheres were collected from both cell lines (Fig. 1c) to assess ZSCAN4 by immunoblot. We found that ZSCAN4 is enriched for in tumorspheres compared with monolayer isogenic cells (Fig. 1d).

### ZSCAN4 marks cells with an enhanced ability to form spheroids

**Fig. 2:**
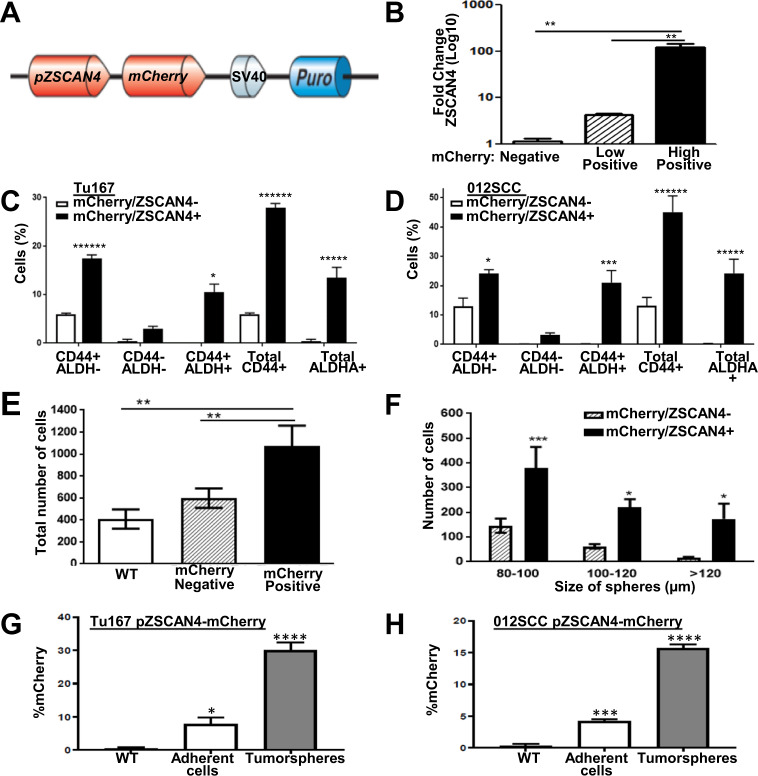
ZSCAN4 expression correlates with CSC markers, larger tumorspheres, and is upregulated in spheroid conditions. **a** A schematic illustration of the lentiviral vector with mCherry reporter under the ZSCAN4 promoter. **b** qRT-PCR for ZSCAN4 expression in Tu167 pZSCAN4-mCherry cells after FACS sorting into three groups: high mCherry (High-Positive), low (Low-Pos), and negatively sorted, indicating ZSCAN4 correlates with mCherry expression. **c** mCherry reporter assay in Tu167 and **d** 012SCC cells indicates that mCherry/ZSCAN4 correlates with the CSC Markers CD44 and ALDH1A1. **e** Tumorsphere formation assay shows a total increase in the number of tumorspheres in mCherry/ZSCAN4 positive cells compared with negative and Tu167 wild type (WT) cells. **f** Classification of tumorspheres according to size demonstrate a major increase in the larger tumorspheres. All data shown as mean ± S.E.M. observed in triplicate in at least three independent experiments (Tu167, with consistent results in 012SCC). **g** pZSCAN4-mCherry Tu167 and **h** 012SCC cells in monolayers (adherent) and 8 days after tumorsphere formation indicates an increase in the frequency of mCherry in tumorspheres. All data shown are mean ± SEM. Asterisks indicate: *p < 0.05, **p < 0.01, ***p < 0.001, ****p < 0.0001, *******p < 10^−7^.

Previous studies in mouse ES cells have shown that m*Zscan4* is transiently expressed in a small fraction of cells in culture at a given time. However, with time, m*Zscan4* expression is gradually activated in all cells [14]. Furthermore, we recently published that the human ZSCAN4 protein is transient and cleared by the proteasome system [30]. To study *ZSCAN4* expression in HNSCC cells, we designed a plasmid containing the mCherry reporter gene under the putative promoter of *ZSCAN4* (pZSCAN4-mCherry) and a Puromycin selection gene. Following sequence verification, we generated a lentiviral vector (Fig. 2a) and stably transduced two separate lines (Tu167 and 012SCC) to generate pZSCAN4-mCherry cells. Fluorescence activated cell sorting (FACS) allowed us to collect mCherry negative cells and compare them to low and high mCherry expressing cells. Our real time qRT-PCR analysis of *ZSCAN4* in the sorted cells validates a positive correlation between mCherry and *ZSCAN4* expression levels (Fig. 2b).

To determine the relationship between ZSCAN4/mCherry and previously reported HNSCC CSC markers, we co-stained pZSCAN4-mCherry cells (both Tu167 and 012SCC) with ALDH1A1 and CD44 and performed a flow cytometry assay. We then analyzed the co-staining in mCherry positive (mCherry+) and negative (mCherry-) cells (Fig. 2c, d; Supplementary Fig. 1a, b). We found that the total number of mCherry+ expressing CD44 is 4.72-fold higher (*p* < 10^−7^) than mCherry- cells in Tu167 and 3.45-fold higher in 012SCC (*p* < 0.0001). Importantly, the average number of Tu167 mCherry+ expressing ALDHA1 was 30.9-fold in Tu167 and 80.77-fold higher in 012SCC cells (Fig. 2c, d; Supplementary Fig. 1a, b), as the majority >99% of mCherry- (Tu167, 012SCC) cells were ALDHA1 negative. Remarkably, 10.49 ± 1.65% of the Tu167 (*p* < 00.001) and 21.05 ± 4.1% of 012SCC (*p* < 0.001) mCherry+ were triple positive (Fig. 2c, d; Supplementary Fig. 1a, b), suggesting the use of ZSCAN4 as a CSC marker may further identify the CSC population.

To assess the effect of *ZSCAN4* on tumorsphere formation, we FACS sorted our Tu167 and 012SCC pZSCAN4-mCherry cells to mCherry negative and positive cells and performed a tumorsphere formation assay (Supplmentary Fig. 2a, b). Our data indicate that high mCherry/ZSCAN4 positive cells show significantly enhanced ability to form tumorspheres when compared with WT cells (*p* < 0.01) and mCherry negative control cells (*p* < 0.01) (Fig. 2e). Positive mCherry expression also correlates with increased spheroid size (Fig. 2f). Consistent with the upregulation of *ZSCAN4* observed in tumorspheres, we noted that the negatively sorted cells reactivate mCherry in the tumorsphere conditions (Supplementary Fig. 2C). These findings support the idea that ZSCAN4 expression marks and correlates with an enhanced ability to form spheres.

Next, we sought to find whether the upregulation of ZSCAN4 in tumorspheres represents an increase in the number of cells positive for ZSCAN4 and to find if ZSCAN4-negative cells can generate ZSCAN4-positive cells in monolayer culture. Therefore, we performed a tumorsphere assay with pZSCAN4-mCherry cells (Tu167, 012SCC), and compared the frequency of mCherry in adherent cells versus spheroids by flow cytometry (Fig. 2g, h). Our results indicate a significant enrichment (*p* < 0.0001; Tu167, 012SCC) in the frequency of mCherry positive cells in tumorspheres compared with attached cells. Conversely, FACS sorting of mCherry positive and negative cells in adherent condition followed by growing the cells in monolayer again, results in the reduction of mCherry positivity to nearly the starting frequency, while only 0.4% of the negative cells have reactivated mCherry (Supplementary Fig. 2C). These data validate that spheroid conditions increase ZSCAN4 positive state frequency and suggest its transient expression.

### ZSCAN4 induction increases the frequency of tumorspheres in vitro and CSCs in vivo

We previously reported that ZSCAN4 is cleared from the cells by the proteasome system [30]. To determine the effect of ZSCAN4 in HNSCCs, and to allow us to trigger it transiently, we generated cell lines in which ZSCAN4 is induced in response to Doxycycline (Dox) (Tu167 and 012SCC tet-ZSCAN4 cells) (Supplementary Fig. 3a–c). Our data show that upon addition of Dox to the medium, ZSCAN4 is detectable within 6 h (Supplementary Fig. 3d). We verified the transient induction of ZSCAN4, by treating the cells for 48 h, and then removed Dox to follow ZSCAN4 protein clearance. Our data indicate that 48 h following Dox removal, ZSCAN4 is depleted from the cells (Supplementary Fig. 3e).

**Fig. 3:**
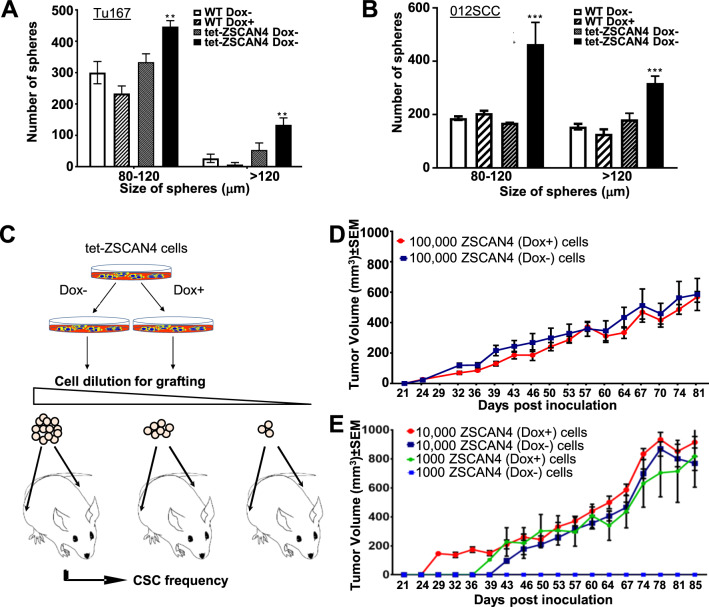
ELDA shows ZSCAN4 induction increases the frequency and size of tumorspheres and tumors. **a** Induction of ZSCAN4 in tet-ZSCAN4 Tu167 cells and **b** 012SCC cells by addition of doxycycline for 48 h (Dox+), prior to generation of tumorspheres, significantly increases the number and size of tumorspheres compared with untreated isogenic controls (Dox−) and to wild type (WT) treated or untreated with Dox. The statistical significance between the groups was determined using two away ANOVA with multiple Tukey’s post hoc comparisons. Asterisks indicate a significant difference from isogenic untreated and WT cells: **p < 0.01, ***p < 0.001. **c** Illustration of Extreme limiting dilution assay (ELDA) in vivo in NGS immunodeficient mice. tet-ZSCAN4 (Tu167) cells were treated (Dox+) or untreated (Dox−) with Dox for 48 h in culture. No Dox was given to the mice throughout the rest of the experiment. Cells were injected subcutaneously into the right and left flank of NOD/SCID gamma immunodeficient mice in multiple increasing dilutions: 100,000 cells, 10,000 cells, and 1000 cells per inoculation (*n* = 8), and allowed to form tumors for up to 85 days. **d**, **e** Tumor growth shown are mean ± SEM for each group (100,000 *n* = 6; 10,000 *n* = 8; 1000 *n* = 8).

Then, to define the effect of ZSCAN4, we used a pulse of ZSCAN4 induction by incubation of the tet-ZSCAN4 cells (Tu167 and 012SCC) with Dox for 48 h. Untreated (Dox−) cells were used as controls. Cells were then grown as tumorspheres for up to 11 days without Dox. As additional controls for the potential effects of Dox, we used isogenic Empty wild type (WT) cells in the absence (Dox−) or presence of Dox (Dox+). Our data indicate that ZSCAN4 induction significantly increases both the number and size of tumorspheres (Fig. 3a, b).

To determine the effect of ZSCAN4 on tumorgenicity in vivo, we utilized the extreme limiting dilution assay (ELDA) in the immune compromised NSG (NOD/SCID/IL2Rγ−/−) mouse xenograft model [31]. tet-ZSCAN4 (Tu167) cells were treated (Dox+) or untreated (Dox−) with Dox for 48 h in culture and then injected into the right and left flanks of NSG mice at concentrations of 1000 to 100,000 cells and monitored over 85 days (Fig. 3c). No Dox was given to the mice throughout the experiment. When 100,000 cells were inoculated, no significant difference was detected compared with the untreated controls (Dox−) (Fig. 3d). Remarkably however, when 10,000 cells were injected, (Dox+) tumors were measurable within 29 days (*n* = 8) compared with untreated cells which were palpable only after 43 days (*n* = 4), yet tumors measurable only after 60 days (*n* = 7). More strikingly, inoculation of 1000 cells shows that ZSCAN4 induced cells formed tumors as early as 39 days, while the untreated cells formed no tumors (Fig. 3e). Following the termination of the experiment, the frequency of CSC was calculated by L-Calc software (StemCell Technologies) and indicated that ZSCAN4 induction prior to inoculation significantly increases the CSC frequency to 1 in every 721 cells (*p* < 0.0001), whereas only 1 in 4746 control (Dox−) cells had the ability to form tumors (Table 1).

**Table 1:** ELDA assay in vivo show that ZSCAN4 induction significantly increases the frequency of CSC (*p* < 0.0001).

Group	No. of cells/inoculation			CSC frequency (1 in/…) *p* < 0.0001
	100,000	10,000	1000	
**tet-ZSCAN4 Dox−**	6/6	7/8	0/8	4746
**tet-ZSCAN4 Dox+**	6/6	8/8	6/8	721

Tu167 tet-ZSCAN4 cells were treated (Dox+) or untreated (Dox−) for 48 h and then engrafted into NGS immune-deficient mice. Mice were not treated with Dox thereafter. The development of xenografts was assessed up to 85 days post inoculation. Frequencies of CSC were calculated using L-Calc software.

CSCs are defined by their ability to form secondary tumors upon injection of cells at extremely low cell numbers into immune-deficient mice. Therefore, to determine the effect of in vivo ZSCAN4 induction on CSC frequency, primary tumors were made in NSG mice (*n* = 8). To induce ZSCAN4 in vivo, a day after inoculation, a solution of 200 µg/ml doxycycline in 5% sucrose was added to drinking water (Dox+) for 48 h to one group (*n* = 4), while only 5% sucrose was given to the untreated control group (Dox−) (*n* = 4). Mice were allowed to develop tumors and kept thereafter without Dox for an additional 5 weeks. Next, for in vivo ELDA, tumors were excised, and mice were inoculated with 100,000, 10,000, or 1000 dissociated tumor cells (*n* = 10 per group) to generate secondary tumors and monitored daily for 7 weeks. Although the in vivo ZSCAN4 induction event by Dox was temporally remote (total of 12 weeks), our ELDA indicate a significant 2.5-fold increase (*p* < 0.001) in CSC frequency in vivo (Supplementary Fig. 4). These data suggest a long-lasting effect for ZSCAN4 on CSC frequency.

### ZSCAN4 promotes stem cell factor expression

**Fig. 4:**
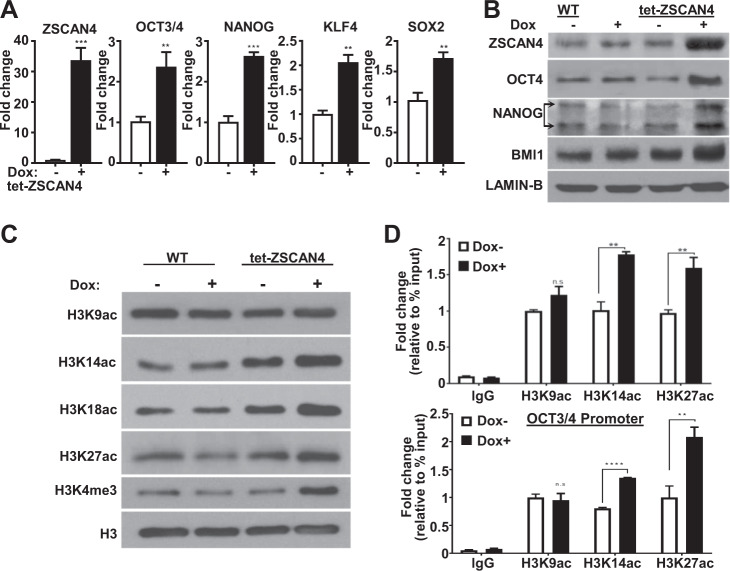
Induction of ZSCAN4 promotes CSC factor expression and facilitates chromatin remodeling. ZSCAN4 induction (tet-ZSCAN4 Tu167 cells) results in a significant increase in **a** pluripotency factor expression (OCT4, NANOG, KLF4, and SOX2) as shown by qRT-PCR. Data shown as mean ± S.E.M. observed in triplicate in three independent experiments. Separate *t*-tests confirm a significant difference from isogenic untreated cells: **p < 0.01, ***p < 0.001. **b** immunoblots show a marked increase in pluripotency and CSC markers. **c** Immunoblot analyses indicate ZSCAN4 induction leads to an increase in open chromatin marks: histone 3 (H3) acetylation at lysine residues 14, 18, and 27 (K14ac, K18ac, K27ac) and H3K4 methylation (H3K4me). H3 was used as a loading control. **d** Chip-qPCR indicates a significant enrichment in histone 3 acetylation at Lysine 14 and 27 at NANOG and OCT3/4 promoters after ZSCAN4 induction. Data shown as mean ± S.E.M. The statistical significance between the two groups was determined by separate *t*-tests ***p < 0.01. ****p < 0.0001.

CSCs harness stem-cell related mechanisms to improve survival and have been reported to display higher expression levels of the core pluripotency genes OCT3/4, NANOG, KLF4, and SOX2 [5, 7, 10–12, 32]. Furthermore, high expression of these transcription factors has been shown to mark CSCs and promote their survival and self-renewal [7, 33–35]. We therefore tested the effect of ZSCAN4 on the core pluripotency genes in our tet-ZSCAN4 cell lines (Tu167 and 012SCC cells). Our data by qRT-PCR indicate that ZSCAN4 induction for 48 h leads to significant upregulation of OCT3/4 (*p* < 0.01), NANOG (*p* < 0.0001), KLF4 (*p* < 0.01), and SOX2 (*p* < 0.01) (Fig. 4a). The increase in CSC factors was further validated by immunoblot (Fig. 4b) and by immunostaining with HNSCC CSC markers BMI1 and CD44 (Supplementary Fig. 5). These findings are important as these factors directly regulate the expression of tumor stemness and proliferation genes [36, 37] and suggest that ZSCAN4 promotes the upregulation of pluripotency and CSC markers.

### ZSCAN4 induction facilitates chromatin remodeling at pluripotency gene promoters

To accommodate the transcriptional programs that maintain pluripotency, ES and iPSC adopt an open chromatin state [26]. Interestingly, murine *Zscan4* expression has been found to correlate with more efficient nuclear reprogramming during generation of iPSC, as well as with heterochromatin de-condensation marks in mES cells, specifically with histone hyperacetylation [21, 38]. Still, it remained unclear if ZSCAN4 was involved in this process. To determine if expression of human ZSCAN4 leads to similar epigenetic changes in cancer cells, we examined a panel of acetylation patterns of Histone 3 Lysine residues 9, 14, 18, and 27 (H3K9ac, H3K14ac, H3K18ac, and H3K27ac) after ZSCAN4 induction. We show by immunoblot analysis that ZSCAN4 induction for 24 h leads to significantly elevated histone 3 hyperacetylation, particularly on Lysine residues 14, 18, and 27 (Fig. 4c).

To determine the functional relation between ZSCAN4, H3 hyperacetylation and pluripotency gene upregulation, we performed chromatin immunoprecipitation (ChIP) assays with antibodies specific to H3K14ac and H3K27ac following 24 h of ZSCAN4 induction. We also included the antibody for H3K9ac as a control, as this histone acetylation mark remained at comparable levels after ZSCAN4 induction. Our data confirm the induction of ZSCAN4 leads to histone 3 hyperacetylation at NANOG and OCT3/4 promoters (Fig. 4d), suggesting its role in chromatin de-condensation and promoting CSC factor expression.

### ZSCAN4 is required for maintenance of the CSC phenotype

HNSCC CSCs are marked by high expression of the surface maker CD44 [9, 12, 28, 36] and the polycomb repressive complex members EZH2 and BMI1 [9, 37, 39, 40]. EZH2 has been shown to be enriched in HNSCC where it is required for CSC survival [41, 42]. BMI-1 is upregulated in multiple cancers where it promotes the CSC phenotype and correlates with poor prognosis [37, 39, 40]. In addition, high expression of the pluripotency factors OCT3/4, NANOG, and SOX2 also mark CSCs [5–7]. To determine if ZSCAN4 is needed for the maintenance of CSC, and CSC marker expression, we first used pU6-ZSCAN4 shRNA knockdown vector (Origene) (containing RFP reporter gene and puromycin resistance gene) (Supplementary Fig. 6a). We then tested the knockdown efficiency of four ZSCAN4 shRNA sequences (named shRNA1–shRNA4) by transfection into Tu167 HNSCC cells. As controls, we used scrambled non-targeting control shRNA (NTC-shRNA), and Empty vector (same vector without an shRNA sequence). Our data by reverse transcription qPCR (Supplementary Fig. 6b), and immunostaining (Supplementary Fig. 6c), confirm that all four shRNA sequences efficiently downregulate ZSCAN4 expression.

**Fig. 5:**
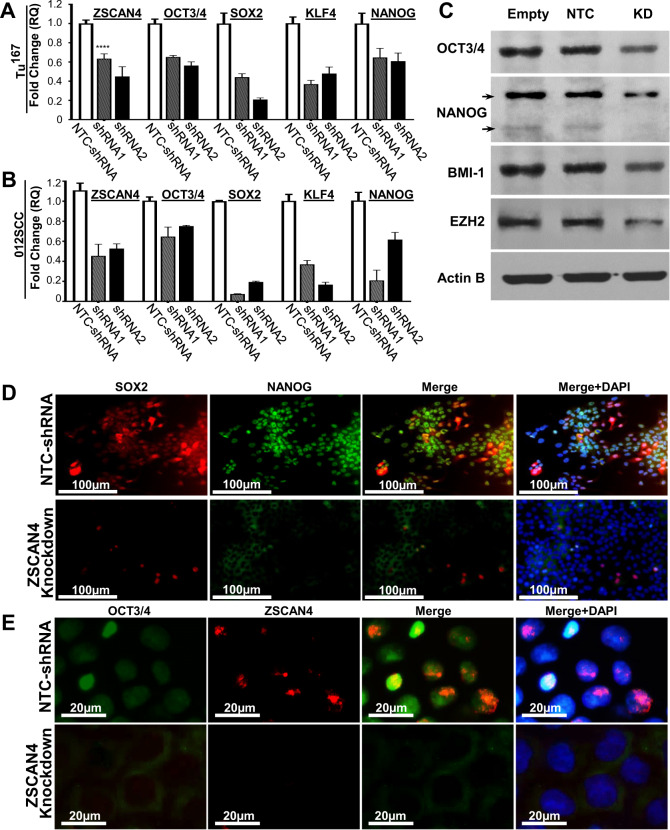
ZSCAN4 is required for the expression of cancer stem cell markers. RT-qPCR analysis of ZSCAN4 knockdown (KD) by two different shRNA (shRNA 1 and 2) in Tu167 (**a**) and 012SCC (**b**), indicates that ZSCAN4 depletion results in decreased expression of the pluripotent stem cell factors OCT3/4, SOX2, KLF4, and NANOG compared with non-targeting control (NTC) shRNA in isogenic control cells. Asterisks indicate: *p<0.05, **p<0.01, ***p<0.001. The statistical significance was determined by two away ANOVA with multiple Tukey’s post hoc tests. The reduction in pluripotency and CSC factors were further validated by: **c** Immunoblot after ZSCAN4 knockdown compared to isogenic cells with Empty vector or NTC-shRNA expressing endogenous levels of ZSCAN4. Actin B was used as loading control. **d** Representative images of co-immunostaining of SOX2 (red) and NANOG (green) as well as **e** OCT3/4 (green) and ZSCAN4 (red). Nuclei are visualized by DAPI.

Next, we used two of the shRNA sequences (shRNA1 and shRNA2) to generate stable knockdown cell lines (in Tu167 and 012SCC cells). Isogenic cells for each cell line with NTC-shRNA, or empty vector were used as controls. Consistent with our finding that pluripotency factors are upregulated by ZSCAN4 induction (Fig. 4a, b), our results by qPCR assay in Tu167 (Fig. 5a) and 012SCC cells (Fig. 5b), indicate that ZSCAN4 depletion by two different shRNA (shRNA1 and 2) results in significant downregulation of OCT3/4, SOX2, KLF4, and NANOG. These data were further corroborated by immunoblot (Fig. 5c) and immunostaining (Fig. 5d, e). Our results further indicate a decrease in the CSC markers BMI1 and EZH2 (Fig. 5c). These data suggest that depletion of ZSCAN4 may alter HNSCC CSC potency.

**Fig. 6:**
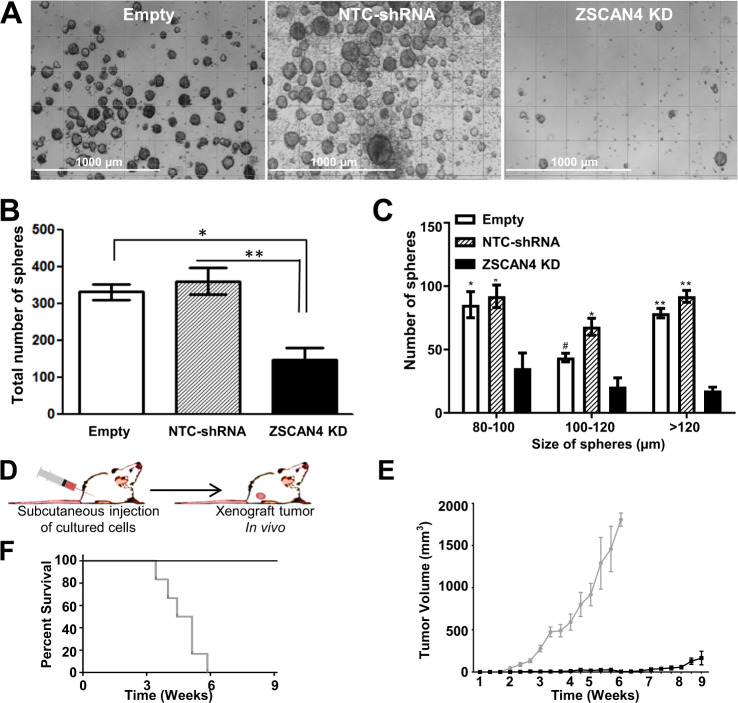
ZSCAN4 is essential for tumorsphere growth and survival and depletion severely affects tumor growth. **a** Representative images of tumorspheres in ZSCAN4 depleted cells compared with isogenic cells with Empty vector or NTC-shRNA. Scale bar indicate 1000 µm. **b** ZSCAN4 Knockdown (KD) results in fewer and **c** smaller tumorspheres when compared with both control cell lines (Empty and NTC-shRNA). Significance of data were confirmed by separate one-way ANOVAs followed by Tukey’s post hoc tests. All data shown as mean ± S.E.M. observed in triplicate in at least three independent experiments. Asterisks indicate: *p < 0.05, **p < 0.01. **d** Schematic illustration of mouse xenograft model. NGS mice were injected subcutaneously with Tu167 ZSCAN4 knockdown cells (*n* = 10), or NTC-shRNA cells as controls (*n* = 10) and allowed to form xenograft tumors. **e** Tumor volume at indicated time. Error bars denote S.E.M., (*p* ≤ 0.001) starting from week 3. **f** Kaplan–Meier survival curve of mice inoculated (*p* ≤ 0.001); results are shown from day of cell injection to the day of euthanasia.

To determine if ZSCAN4 is necessary for spheroid formation, we performed tumorsphere formation assays in ZSCAN4 knockdown cell lines, the control isogenic NTC-shRNA, and Empty vector cells (Fig. 6a). Our results indicate that ZSCAN4 depletion leads to a dramatic reduction in the overall number (Fig. 6b) (*p* < 0.01), and size of spheroids compared with both NTC-shRNA and Empty vector control cells (Fig. 6c) (*p* < 0.01). Collectively, our data suggest that ZSCAN4 is essential for the maintenance of HNSCC CSCs and CSC factors.

### ZSCAN4 depletion severely affects tumor growth

To assess the potential of ZSCAN4 as a therapeutic target in cancer, we assessed the impact of ZSCAN4 depletion on tumor growth in vivo using the NSG [31] mouse xenograft model. We subcutaneously injected either 1 × 10^6^ freshly generated ZSCAN4 knockdown cells (Tu167) or 1 × 10^6^ isogenic non-targeting control NTC-shRNA cells into the flanks of female NSG mice (Fig. 6d). As expected, a large tumor develops in all the control mice injected with NTC-shRNA cells leading to the need for euthanasia by 5 weeks (Fig. 6f). Importantly, our data indicate that ZSCAN4 depletion results in a significant inhibition of more than 98% in tumor growth and only half of the mice present with a palpable tumor after 9 weeks. We show that ZSCAN4 knockdown inoculated mice survive (Fig. 6f) as the tumors remain significantly attenuated even at the 9-week endpoint (Fig. 6e, f). These data suggest that ZSCAN4 is required for tumor growth and development.

## Discussion

HNSCC remains a growing public health concern and the current incidence in oropharyngeal HNC has reached epidemic rates [43, 44]. Late-stage HNSCC, constituting over 60% of HNSCC cases, remains difficult to treat [45, 46]. For locally advanced and metastatic HNSCC, multi-modal treatment involving combinations of surgery, radiotherapy, or chemotherapy have become the standard of care [45]. Unfortunately, these therapies have not significantly improved survival, with treatment failure attributed to tumor recurrence and metastasis [47]. Therefore, new therapeutic approaches are needed. Previous studies have reported a population of CSCs within HNSCC tumors [48], capable of differentiation to other cell types within the tumor [49], thereby contributing to cancer recurrence, and treatment resistance.

ZSCAN4 has a major role in the maintenance of pluripotent stem cells [16, 19, 21, 50, 51]. Like ZSCAN4, embryonic factors OCT3/4, SOX2, and NANOG are reactivated in cancer and have further been classified as CSC markers that regulate self-renewal and contribute to tumor aggressiveness and metastasis. Previous findings in mES cells, show that the ZSCAN4 expression correlates with histone hyperacetylation [38]. However, it remained unclear whether the human ZSCAN4 is involved in cancer, or if it drives or marks cells with open chromatin states. DNA methylation and hypomethylation are mechanisms for regulation of chromatin state. Due to their extensive role in CSCs and tumorigenesis, inhibitors of chromatin and DNA modifying enzymes like DNMTs, HDACs, and EZH2 are currently used in the clinic [52, 53]. The mZSCAN4 was shown to sequester DNMT1, and consequently to lead to a global DNA hypomethylation in mES cells [50]. However, further studies are needed to investigate whether the human ZSCAN4 has a role in DNA demethylation.

Chromatin remodeling through histone modification is another powerful mechanism for gene regulation. Here we demonstrate for the first time the role of human ZSCAN4 in facilitating chromatin remodeling and the maintenance of HNSCC CSCs. First, we show that ZSCAN4 is enriched in tumorspheres, is co-expressed with CSC markers, and by using a reporter gene under the ZSCAN4 promoter, we demonstrate it marks cells with higher capacity to form spheroids in vitro. These findings suggest that high levels of ZSCAN4 mark the HNSCC CSCs. Second, our studies indicate that ZSCAN4 facilitates CSC marker expression. Upon a short induction of ZSCAN4, CSC markers including BMI1, CD44, and the core stem cell factors OCT3/4, SOX2, NANOG, and KLF4 are significantly upregulated, while depletion of ZSCAN4 results in a significant downregulation of these factors. Third and importantly, we define for the first time a role of ZSCAN4 in upregulation of histone H3 acetylation and show its epigenetic effect on pluripotency gene promoters. Our data indicate that ZSCAN4 leads to a functional histone 3 hyperacetylation at the promoters of OCT3/4 and NANOG and to an elevated expression of OCT3/4, SOX2, NANOG, and KLF4. Fourth, our tumorsphere ELDA as well as two in vivo ELDA assays after ZSCAN4 induction, either prior to or post-inoculation, also suggest an epigenetic effect of ZSCAN4, as ZSCAN4 is depleted from the cells within 48 h after Dox removal (Figs. 3, 4 and Supplementary Fig. 3E), yet remarkably, we found that ZSCAN4 induction has a long-lasting effect on the frequency of CSC. Lastly, we show that ZSCAN4 depletion by gene knockdown leads to a reduction in open chromatin marks, accompanied by downregulation of pluripotency factors and the CSC markers BMI1 and EZH2. Moreover, ZSCAN4 depletion results in reduced ability to form spheroids in vitro and severely affects xenograft tumor growth in vivo. Overall, our findings suggest that ZSCAN4 marks, promotes, and maintains the most highly tumorigenic population of cancer cells.

We previously showed that ZSCAN4 is negatively regulated by the ubiquitin-proteasome system [30]. Given our current data, further research will be required to asses a potential effect of proteasome-ubiquitin modifying drugs and HDAC inhibitors on the frequency of ZSCAN4 and CSCs. While the current acceptable treatment paradigms for HNSCC include surgery with or without radiation therapy or chemotherapy, our data suggests that additional targeted inhibition of ZSCAN4 may function synergistically to enhance treatment efficacy.

## Materials and methods

### Cell lines and cell culture

HNSCC cell lines were authenticated and tested free of mycoplasma by the University of Maryland translational core facility. All cell lines were cultured in complete DMEM medium (Invitrogen) supplemented with 10% fetal bovine serum (Atlanta Biologicals), 2 mM GlutaMAX, penicillin (100 U/mL), and streptomycin (100 μg/mL) (ThermoFisher Scientific).

### RNA extraction and quantitative reverse transcriptase polymerase chain reaction (qRT-PCR)

One microgram of total RNA, isolated with Trizol, was reverse transcribed by Superscript III (Invitrogen) following the manufacturer’s protocol. For qPCR, 10 ng cDNA was used per well in triplicate using SYBR green reaction mix (Roche) following the manufacturer’s instructions with corresponding primers (Supplementary Table 1). Reactions were run on the LightCycler 480 system (Roche). Fold induction was calculated by the delta–delta Ct method.

### Generation of pZSCAN4-mCherry cells

The genomic region containing the ZSCAN4 promoter (2.5 Kb upstream of transcription start codon, and 299 base pairs downstream) was cloned into a lentiviral vector (pEZX- LvPM02; GeneCopoeia) in which the mCherry reporter gene and a puromycin resistance gene are downstream to the putative ZSCAN4 promoter.

### Immunoblot assays

Cytosolic proteins were extracted by Cytoskeleton buffer (10 mM PIPES, 300 mM sucrose, 100 mM NaCl, 3 mM MgCl_2_, 1 mM EGTA, and 0.5% Triton X100), and nucleoplasm fraction was isolated. Next, pellets were lysed in urea solution (8 M Urea in 0.01 Tris pH 8 + 0.1 M NaH_2_PO_4_) and sonicated. Samples were electrophoresed in polyacrylamide gels. PVDF membranes were blocked with 5% nonfat milk or Superblock (Thermo Fisher Scientific) for 1 h, then incubated overnight at 4 °C with the indicated primary antibodies in blocking solution (Supplementary Table 2). Membranes were washed and incubated with secondary antibodies (1:5000) for 1 h. Secondary antibodies were visualized by ECL Chemiluminescence (ThermoFisher Scientific).

### Tumorsphere formation assay

Single cells were harvested using accutase (EMD Millipore) and tumorsphere growth was performed in DMEM F12 (Invitrogen), supplemented with B27 serum replacement (Invitrogen), 20 ng/ml basic FGF (Affymetrix, eBioscience), 20 ng/ml recombinant human EGF (Biolegend), and 100 µg/ml Ampicillin (American Bioanalytical). Tumorspheres were maintained in ultra-low attachment dishes for up to 11 days. Size and number of spheres were analyzed using ImageJ software.

### Immunofluorescence staining

Cells were fixed in 4% PFA and antigen retrieval was performed at 90 °C. Slides were blocked in 1% BSA, 10% fetal bovine serum, and 0.2% Tween 20, and incubated at 4 °C overnight with the primary antibodies anti-NANOG (1:1000, Cell Signaling), anti-ZSCAN4 (1:1000, Origene), anti-OCT3/4 (1:250, Santa Cruz Biotechnology), anti-BMI1 (1:1000), anti-SOX2 (1:500) (Cell Signaling), in blocking solution. The FITC-conjugated anti-CD44 (1:100, Invitrogen) was incubated for 1 h on ice in blocking solution. Nuclei were stained with DAPI (Roche Life Sciences). Uninduced cells (Dox−) and cells stained without primary antibody were used as controls. Samples were visualized with fluorescent Alexa546, Alexa488 or Alexa647 secondary antibodies (Invitrogen) under a Zeiss 510-confocal microscope.

### CSC markers profiling by flow cytometry

Cells (pZSCAN4-mCherry) were fixed in 4% paraformaldehyde (Alfa Aesar) in DPBS and co-stained for 1 h on ice with the appropriate antibodies: EFLUOR450-conjugated anti-CD44 (1:100), Alexa 647-conjugated anti-ALDH1A1 (1:100). Samples were washed and analyzed by flow cytometry (FACS Canto II; BD Biosciences) and data was generated using FCS Express 7 software.

### Chromatin immunoprecipitation

ChIP was completed with 2.5 million cells/reaction following the Pierce Magnetic ChIP protocol (ThermoFisher Scientific). Sheared chromatin was immunoprecipitated with indicated antibodies (Supplementary Table 2). The provided anti-RNA Polymerase II (1 µg) (not shown) and a Rabbit IgG (10 µg) were used as additional controls. qRT-PCR primers are shown in Supplementary Table 3.

### Extreme limiting dilution (ELDA) and tumorigenicity in NSG mice

Both ELDA in vivo experiments were performed in the Translational Laboratory Shared-Services and conformed with the guidelines of the Institutional Animal Care and Use Committee (IACUC protocol #1016012). tet-ZSCAN4 (Tu167) cells were induced with Dox for 48 h (Dox+) in culture, or remained untreated (Dox−), followed by harvesting, counting, and inoculation of the desired cell dose (*n* = 8/dose) into both flanks of immune compromised NSG (NOD.Cg-*Prkdc*^*scid*^ *Il2rg*^*tm1Wjl*^/SzJ) [31] female mice (2 months old; Charles River). Mice were monitored over 85 days, and tumors were measured by caliper biweekly. Assessments were done as double-blind assay. The CSC frequency was calculated by L-Calc™ Software (StemCell Technologies) (https://www.stemcell.com/l-calc-software.html).

### In vivo and secondary xenograft tumors ELDA assay

All procedures were approved by and performed according to IACUC protocol #1016012. tet-ZSCAN4 (Tu167) cells were diluted to the desired dose and injected into the flank of NSG [31] female mice (Charles River) (*n* = 8). To induce ZSCAN4 in vivo, a day after inoculation, 200 µg/ml doxycycline in 5% sucrose was added to the drinking water (Dox+) for 48 h, while only 5% sucrose was used for the controls (Dox−) (*n* = 4 per group). No Dox was given thereafter. Tumors were measured by a caliper biweekly. For in vivo ELDA, mice were sacrificed, tumors were minced, dissociated with 1.6 U/ml Liberase (Roche), 100 ug/ml DNAase (Sigma) and incubated for 60 min at 37 °C. Mice were injected with 1000, 10,000, or 100,000 tumor cells (*n* = 10/group), and monitored daily. Researchers were blinded to the treatment groups. CSC frequency was calculated by L-Calc™ Software.

### Generation of ZSCAN4 knockdown and control cells

ZSCAN4 shRNAs were tested (19-mer sense, a hairpin loop, and 19-mer anti-sense oligos): shRNA1: 5′-GAGAACGGTCCTAGGCCTGTCAAGAGGAGAACGGTCCTAGGCCT G-3′, shRNA2: 5′-GATATCAGACCTACGGGTGTCAAGAGGATATCAGACCTACGGG TG-3, shRNA3: 5′-CTCGAGTAAATGAAAATATTCAAGAGCTCGAGTAAATGAAAATA T-3 and two were cloned into the HuSH shRNA Plasmid pRFP-C-RS (Origene). Vectors were stably transfected into Tu167 or 012SCC cells by Effectene (QIAGEN) according to the manufacturer’s protocol. As controls, isogenic cell lines were also generated expressing a non-targeting control shRNA (NTC-shRNA) or an Empty vector (same vector without an shRNA cassette). Cell lines were selected with 1 µg/ml Puromycin (ThermoFisher Scientific). Knockdown was confirmed by immunostaining and by qPCR.

### ZSCAN4 knockdown xenograft assay

All procedures were approved and performed according to IACUC protocol no. 0711021. To assess the impact of ZSCAN4 depletion, male or female NSG mice (8–12 weeks old) (Jackson Laboratory, Bar Harbor, ME) were randomized into two groups (*n* = 10 per group) and 10^6^ of ZSCAN4 knockdown or NTC shRNA cells were injected subcutaneously on the left flank of each mouse. Tumor volume based on external caliper measurements were calculated by the formula: Tumor volume = 1/2(length × width^2^). Investigators were blinded to experimental groups and outcome assessments during experiments.

### Statistical analyses

All data are shown as the mean ± S.E.M of multiple independent experiments, with biological replicates. Student’s *t*-test or one-way ANOVAs (when appropriate) were used for statistical analyses. Significant interactions were followed by Tukey or Bonferroni post-hoc comparisons when appropriate. Statistical analyses and figure generation were performed with STATISTICA 13 and GraphPad Prism 5 software. For in vivo experiments, statistical significance of difference in tumor volume was assessed by two-way ANOVA with repeated measures and Tukey’s post hoc comparisons. The mice were randomly assigned to the experimental groups. Investigators were blind to experimental groups and outcome assessments during experiments.

## Supplementary information

Supplemental Figures 1-6

Supplemental Tables 1-3
