# Supplementary material for: ZSCAN4 facilitates chromatin remodeling and promotes the cancer stem cell phenotype
Source: Oncogene. 2020 Jun 7;39(26):4970–82. doi: 10.1038/s41388-020-1333-1 (PMC7314663; doi:10.1038/s41388-020-1333-1)
Supplement: Supplementary file 1 — Supplemental Figures 1-6 [file 41388_2020_1333_MOESM1_ESM.pdf]

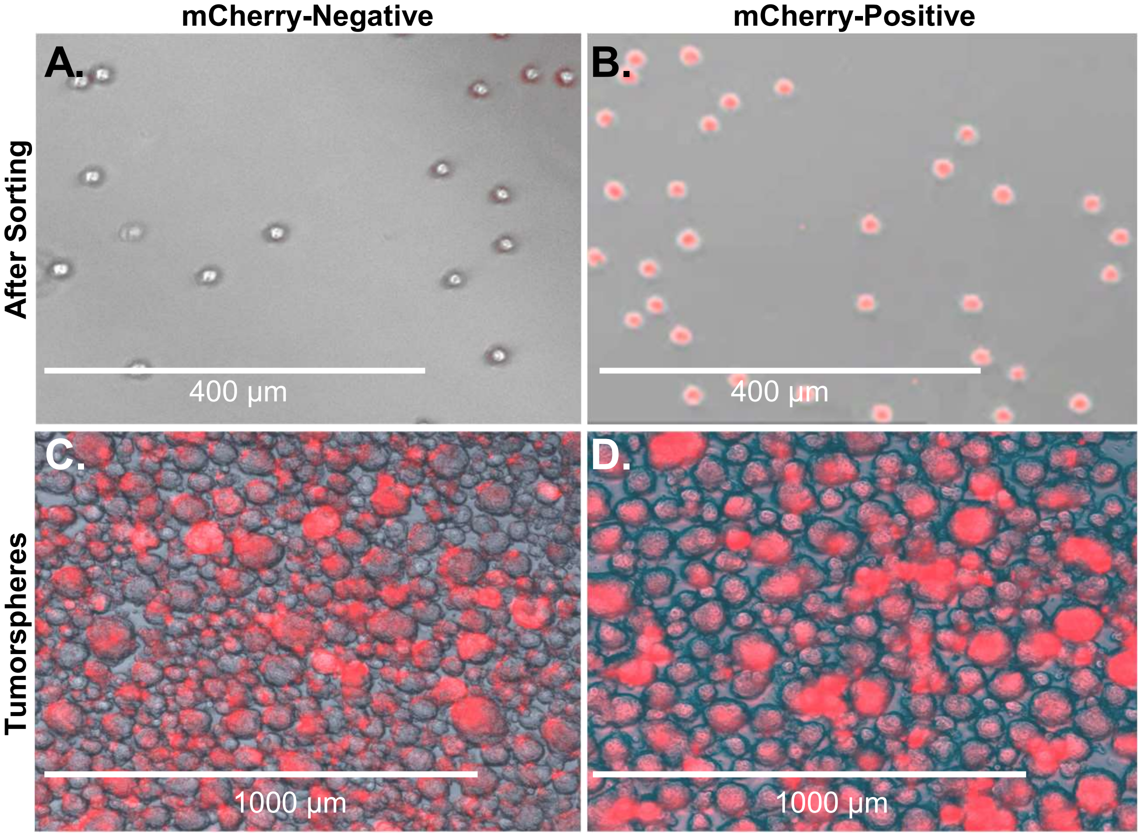


**Supplementary Figure 1. ZSCAN4 marks tumorsphere forming cells**. Representative fluorescent and Phase images of single pZscan4-mCherry cells (Tu167) immediately after FACS sorting and culturing. Size bar = 400 um. The cells were divided into two groups: **A**, mCherry negative and **B**, mCherry positive. **C, D**, Cells were then subjected to spheroid formation assay. Representative images showing ZSCAN4 expression marks cells with higher ability to form tumorspheres. Further, tumorsphere from mCherry negative cells are mCherry positive. Scale bar = 1000 um. Representative images from data obtained in triplicate in at least three independent experiments.

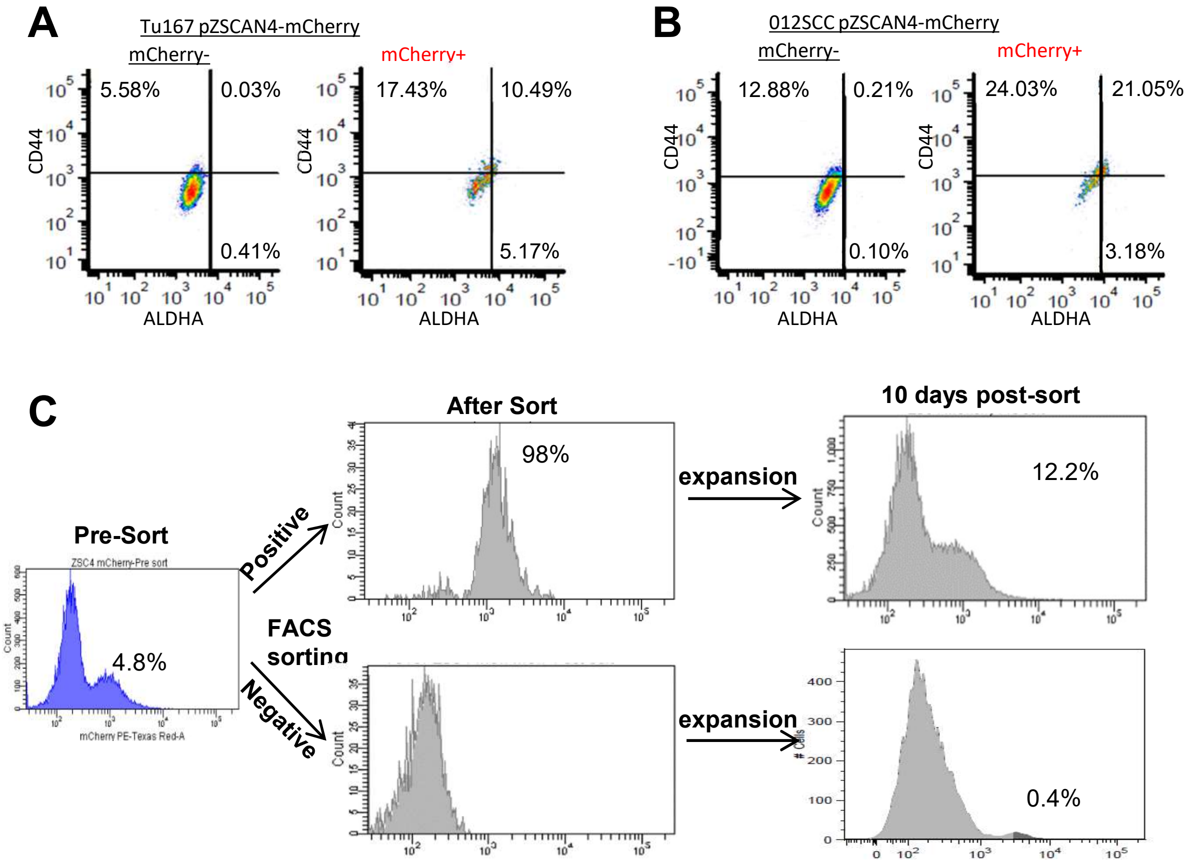


**Supplemental Figure 2. ZSCAN4 expression in HNSCC cells in transient A,** Flow cytometry in mCherry reporter cells pZSCAN4-mCherry Tu167 cells and **B**, 012SCC cells indicates that mCherry/ZSCAN4 correlates with the CSC Markers CD44 and ALDH1A1. **C**, FACS analysis of pZSCAN4-mCherry cells demonstrates that 14.8% are mCherry positive (ZSCAN4 expressing). Then, cells were sorted into 100% mCherry expressing cells and 100% mCherry negative cells and allowed to grow in monolayer again. After 10 days of expansion mCherry positive cells constituted 12.2% of the population, while 0.4% of the mCherry negative became mCherry positive.

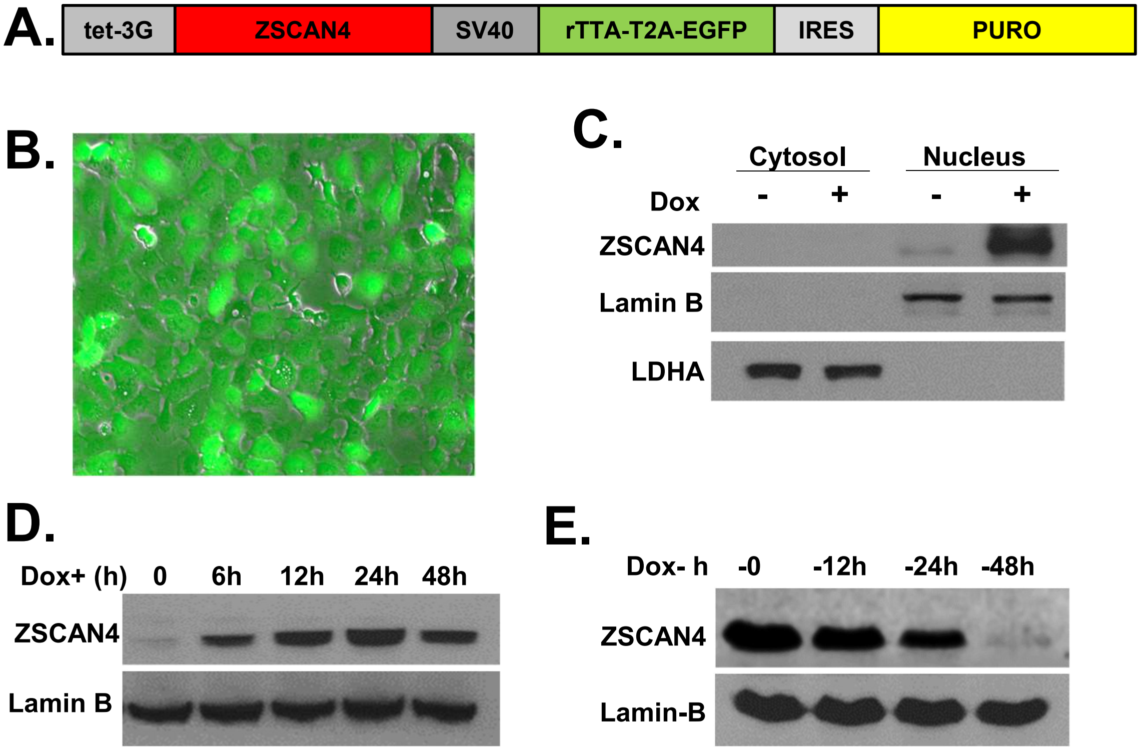


**Supplementary Figure 3**: **A,** An illustration of the tet-ZSCAN4 expression vector. **B,** Image of tet-ZSCAN4 (Tu167) cells showing expression of GFP (green). **C,** Immunoblot analyses indicate ZSCAN4 is tightly regulated by Dox and localizes to the nucleus; Controls: LDH (cytosol), Lamin-B (nucleus). **D,** Doxycycline addition to the culture medium (Dox+) induces ZSCAN4 within 6 hours. **E,** Dox treatment for 48 hours followed by Doc removal (Dox-) leads to clearance of the exogenous ZSCAN4 protein within 48 hours.

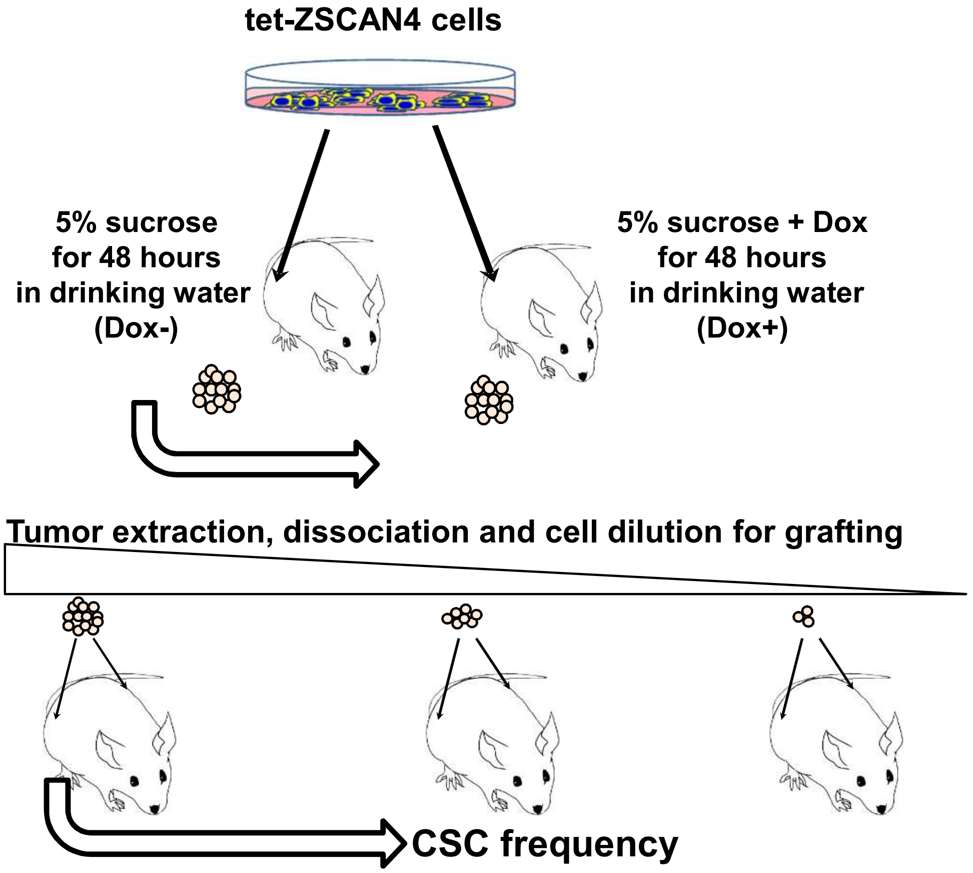


| Group | Cells/Inoculation | | | CSC Frequency P<0.0001 |
|---|---|---|---|---|
| | 100,000 | 10,000 | 1,000 | |
| tet-ZSCAN4 Dox- | 5/5 | 9/10 | 6/10 | 1:2,514 |
| tet-ZSCAN4 Dox+ | 5/5 | 10/10 | 6/10 | 1:1,090 |

**Supplementary Figure 4: ELDA assay following ZSCAN4 induction *in vivo* show that secondary tumors and the frequency of CSC are significantly increased** ($p < 0.001$). One million Tu167 tet-ZSCAN4 cells were engrafted into NGS immune-deficient mice. A day later, the mice were treated (Dox+) or untreated (Dox-) in the drinking water for 48 hours. Mice were not treated with Dox thereafter and xenografts were formed for 37 days. Next, tumors were excised and *in vivo* ELDA was performed without additional Dox treatment, with secondary tumors in the indicated cell concentrations. The development of xenografts was assessed 40 days post inoculation. Frequencies of CSC were calculated using L-Calc software.

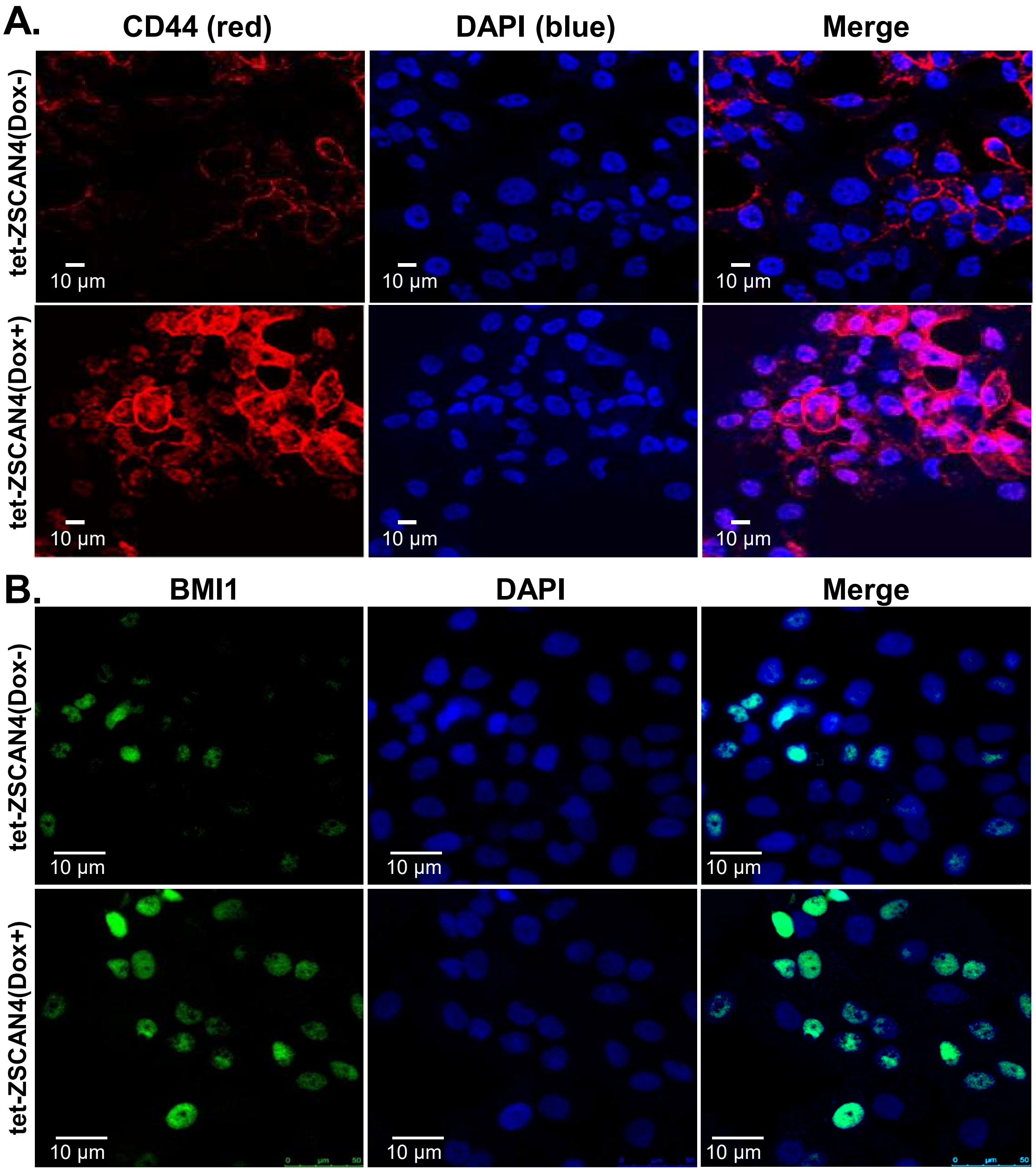


**Supplementary Figure 5: ZSCAN4 induction leads to increase in CSC markers**. Immunostaining of the HNSCC cancer stem cell factors: **A**, CD44 (red) and **B**, BMI1 (green) before (Dox-) and after ZSCAN4 induction (Dox+) indicate a significant increase in CSC marker frequency and intensity. (TU167 cell line). Nuclei are stained with DAPI (blue).

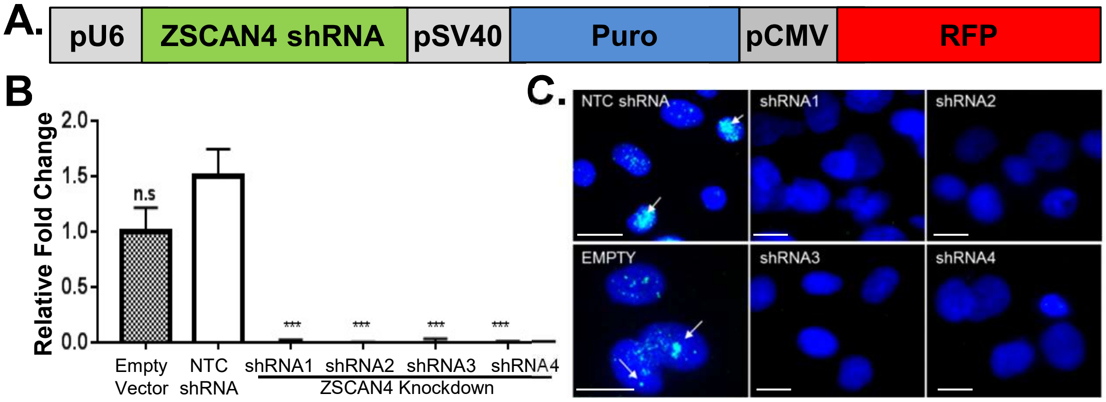


**Supplementary Figure 6. Generation of ZSCAN4 knockdown cells. A**, Illustration of ZSCAN4 knockdown vector used to transfect Tu167 and 012SCC cancer cell lines. **B**, Confirmation of ZSCAN4 knockdown by all four ZSCAN4 shRNA sequences (shRNA1-shRNA4) in Tu167 cells as shown by real-time qRT-PCR analysis. No significant (n.s) effect in ZSCAN4 expression was detected in NTC-shRNA control compared to isogenic cells transfected with Empty vector (same vector with no shRNA). *** Asterisks indicate $p < 0.001$. **C**, Representative images of ZSCAN4 immunostaining (green) in knockdown and isogenic controls. Panel shows nuclei of cells transfected with shRNA1-4. As controls we used cells transfected with Empty vector and cells expressing non-targeting control shRNA (NTC-shRNA). Scale bar = 10 µm. Nuclei are stained by DAPI (blue). Arrows mark ZSCAN4 foci.
