# Supplementary material for: ZSCAN4 facilitates chromatin remodeling and promotes the cancer stem cell phenotype
Source: Oncogene. 2020 Jun 7;39(26):4970–82. doi: 10.1038/s41388-020-1333-1 (PMC7314663; doi:10.1038/s41388-020-1333-1)
Supplement: Supplementary file 2 — Supplemental Tables 1-3 [file 41388_2020_1333_MOESM2_ESM.docx]

| **Marker** | **Forward Primer** | **Reverse Primer** |
| --- | --- | --- |
| ZSCAN4 | 5’-ATCCACCTGCCTTAGTCCAC-3 | 5’-TCGAAGAACTGTTCCAGCCA-3’ |
| RPLP0 | 5’-CAGCAAGTGGGAAGGTGTAATCC-3’ | 5’-CCCATTCTATCATCAACGGGTACAA-3 |
| OCT3/4 | 5’-TCTTCAGGAGATATGCAAAGC-3 | 5’-ATCCTCTCGTTGTGCATAGT-3’ |
| SOX2 | 5’-CAAGGAGAGGCTTCTTGCTGA-3 | 5’-CACAGAGATGGTTCGCCAGT-3 |
| NANOG | 5’-AGCTACAAACAGGTGAAGAC-3’ | 5’-TAGGAAGAGTAAAGGCTGGG-3’ |

**Supplementary Table 1**: List of qRT-PR primers used for expression analyses.

| **Marker** | **Dilution** | **Supplier** |
| --- | --- | --- |
| ZSCAN4 | 1:2000 | Origene |
| OCT3/4 | 1:1000 | BD Transduction Labs |
| EZH2 | 1:1000 | BD Transduction Labs |
| SOX2 | 1:1000 | Abcam |
| NANOG | 1:1000 | Abcam |
| BMI1 | 1:1000 | Abcam |
| H3 | 1:1000 | Cell signaling |
| H3K9ac | 1:1000 | Cell signaling |
| H3K14ac | 1:1000 | Cell signaling |
| H3K18ac | 1:1000 | Cell signaling |
| H3K27ac | 1:1000 | Cell signaling |

**Supplementary Table 2:** List of Antibodies used for immunoblot analyses and ChIP assays.

| **Promoter** | **Forward Primer** | **Reverse Primer** |
| --- | --- | --- |
| pOCT3/4 | 5’-TTACTTAAGTCGACAGAGGTCAGC-3’ | 5’-TGGTCTAGTGCTTGATTCTGTTTG-3' |
| pNANOG | 5’-GAAAGACATGACAAACACCAGAC-3’ | 5’-CAACTAGCTCCATTTT CCTCTTTC-3' |
| Intergenic | 5’-AATGAGTGGGCTCATGGAAA-3' | 5’-TCTGGATGCAGCATTTGTGT-3' |

**Supplementary Table 3**: List of qRT-PR primers for ChIP real-time PCR analyses
